# Low-Temperature Annealing of CdZnTeSe under Bias

**DOI:** 10.3390/s22010171

**Published:** 2021-12-28

**Authors:** Martin Rejhon, Vaclav Dedic, Roman Grill, Jan Franc, Utpal N. Roy, Ralph B. James

**Affiliations:** 1 Faculty of Mathematics and Physics, Charles University, Ke Karlovu 3, 12116 Prague, Czech Republic; rejhonm@gmail.com (M.R.); Roman.Grill@mff.cuni.cz (R.G.); jan.franc@mff.cuni.cz (J.F.); 2 Tandon School of Engineering, New York University, Brooklyn, NY 11201, USA; 3 Savannah River National Laboratory, Savannah River Site, Aiken, SC 29808, USA; Utpal.Roy@srnl.doe.gov (U.N.R.); Ralph.James@srnl.doe.gov (R.B.J.)

**Keywords:** CdZnTeSe, radiation detector, electrodes, low-temperature annealing, space charge

## Abstract

We performed a gradual low-temperature annealing up to 360 K on a CdZnTeSe radiation detector equipped with gold and indium electrodes under bias at both polarities. We observed significant changes in the detector’s resistance and space-charge accumulation. This could potentially lead to the control and improvement of the electronic properties of the detector because the changes are accompanied with the reduction in the bulk dark current and surface leakage current. In this article, we present the results of a detailed study of the internal electric field and conductivity changes in CdZnTeSe detector for various annealing steps under bias taking into account different polarities during annealing and subsequent characterization. We observed that low-temperature annealing results in an increase in the barrier height at the contacts that, in general, reduces the dark current and decreases the positive space charge present in the sample compared to the pre-annealed condition.

## 1. Introduction

CdZnTeSe (CZTS) material has been under development for hard X-ray and gamma-ray semiconductor detectors [1,2,3]. It was observed that the addition of Se to the CdZnTe (CZT) matrix provides improved compositional homogeneity and mitigates many performance-limiting defects [4]. The CZTS material exhibits comparable or even better electrical and spectroscopic properties as CdTe and CZT materials [1,5,6,7,8,9,10].

CdTe-based detectors can be annealed in different atmospheres to improve the electrical and spectroscopic properties. Low-temperature annealing is typically performed on CdTe-based materials in the temperature range of 370–520 K. Studies investigating the influence of low-temperature annealing on CdTe/CdZnTe material have reported controversial results [11,12,13,14,15]. However, these studies were performed on samples with planar electrodes, which do not allow the investigators to distinguish between bulk and surface leakage currents. This is necessary for separating the change in metal/semiconductor interface and surface conditions. Our recent study demonstrated the influence of low-temperature annealing on the surface leakage current and bulk current, especially on the Schottky barrier height [16]. The sample was treated with temperatures between 313 K up to 373 K in ambient air.

Another method used to change the detector properties of CdTe-based materials is electromigration. Here, the charged defects drift in an external electric field. Kim et al. [17] showed the improvement in the mobility-lifetime product after the treatment of CZT at 493 K at a voltage of 60 V for 7 and 60 days, which they assigned to the electromigration of Cu, Fe, and Ga impurities. Additional studies demonstrated the electromigration of positively charged donors in CdTe-based materials, which mainly affect the electrical properties [18,19,20].

In this article, we provide the results of the annealing of CZTS performed at standard operating conditions of detectors to analyze their potential impact on the material and contact characteristics. We performed low-temperature annealing in the temperature range of 320 K to 360 K with an applied bias of ±700 V. We focused on the changes in the electric field distributions and I–V characteristics at room temperature after finishing each annealing step under bias. We observed that low-temperature annealing causes an increase in the barrier height at the contacts that, in general, limits the dark current and decreases the positive space charge present in the sample before annealing.

## 2. Materials and Methods

We studied a CZTS sample with 4% Se and 10% Zn. The sample was cut from a CZTS ingot, which was grown by the traveling heater method with doping of In in the ppm range. The sample dimensions were 5.35×4.60×1.90 mm${}^{3}$. Al${}_{2}$O${}_{3}$ abrasive was used to mechanically polish the sample surface (surface RMS 2 nm) without any further chemical treatment. The metal electrodes were evaporated on the large opposite sides. One contact was planarly made from indium. The opposite gold contact consisted of a guard ring and a central electrode, which allowed us to separate the bulk and surface leakage currents. The area of the central electrode was $2.4\times 2.4\;{\mathrm{mm}}^{2}$. It was separated from the guard ring electrode covering the rest of the surface by a 500 µm wide gap.

**Figure 1:**
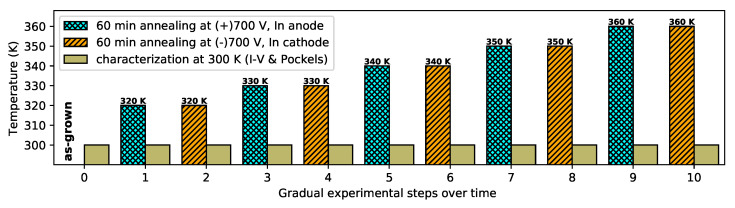
Time scheme of experimental steps. Before the annealing, the as-grown sample was characterized by I–V and the electric field (Pockels) measurements. Each annealing step was conducted in the following way: the sample temperature was stabilized to the required value. Then, a bias of 700 V (with the respective polarity to the indium electrode) was applied for 60 min.

For low-temperature annealing of the CZTS and its characterization, the sample was mounted in an optical cryostat equipped with fine temperature regulation using a Peltier device. An argon atmosphere was maintained in the cryostat at all times. The setup allowed the characterization of the sample between annealing steps without any further manipulation. Before annealing, the as-grown sample was characterized by I–V and electric field (Pockels) measurements (step 0). Each annealing step was conducted in the following way: the sample temperature was stabilized to the required value. Then, a bias of 700 V (with the respective polarity of the indium electrode) was applied for 60 min using a Keithley 2410 source meter. Individual annealing steps were performed for both voltage polarities. The temperature of the annealing steps was increased by 10 K in the range of 320–360 K. Electronic properties were measured after each step at 300 K. A scheme of gradual experimental steps over time is shown in Figure 1.

For the characterization at 300 K, the CZTS sample was biased by a Keithley 2410 source meter, and I–V curves were measured by two Keithley 2000 multimeters (bulk and leakage currents) in the range of ±800V. The distributions of internal electric fields between electrodes were measured by a standard cross-polarizers technique using the electro-optic Pockels effect for the CZTS crystal, which has a $\bar{4}3$ m symmetry [21]. This method allows evaluating a spatial distribution of the internal electric field E(x,y) of a biased sample at ±800V from the relative transmittance distribution $T\left(x,y\right)=I\left(x,y\right)/I_{0}\left(x,y\right)$ measured using low-intensity light (LED at 1550 nm) and an infrared InGaAs CMOS camera. Here, I(x,y) is the passed light intensity distribution of the biased sample with perpendicular polarizers, and $I_{0}\left(x,y\right)$ is the passed light intensity distribution of the sample at zero bias with parallel polarizers. Then, $E\left(x,y\right)∼\arcsin\sqrt{T(x,y)}$.

## 3. Results and Discussion

The analysis of the influence of low-temperature annealing under bias on the CZTS detector is based on the study of changes in the total space charge, profiles of the internal electric fields, and I–V curves between individual annealing steps.

### 3.1. Electric Field and Space Charge

**Figure 2:**
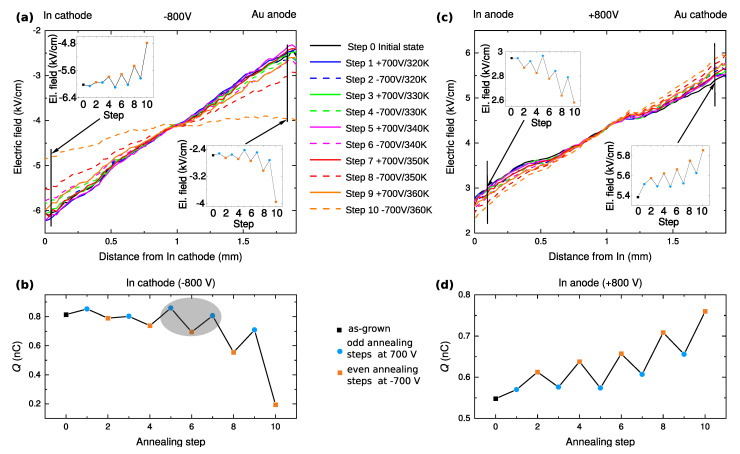
(**a**) The internal electric field profiles, when In contact acts as a cathode and Au contact acts as an anode at room temperature (T=300 K). Two insets show a dependency of the electric field near the contacts. (**b**) Corresponding total space charge in the sample. (**c**) The electric field profiles when a positive voltage is applied to the In contact at room temperature (T=300 K). Here, the data points from the grey area were later used for detailed analysis of the physical model in Section 3.3. (**d**) Calculated total space charge in the sample.

The internal electric field distributions measured after each annealing step are depicted in Figure 2a,c for −800 V and +800 V, respectively. Here, and in all other cases, the polarity is always related to the indium electrode, i.e., the positive/negative polarity means that the indium contact is anode/cathode. The total space charge *Q* was calculated from the electric field distribution [22] as $Q=\varepsilon S(E_{C}-E_{A})$, where $\varepsilon =10.3\varepsilon_{0}$ is the permittivity, *S* is the electrode area, and $E_{C}$ and $E_{A}$ are the electric field amplitudes at the cathode and the anode, respectively. The corresponding total space charge for each step and both polarities is shown in Figure 2b,d.

In the as-grown sample, the absolute value of the electric field (black solid line) decreases from the cathode to the anode for both polarities. It testifies to the presence of a positive space charge in the sample (black square in Figure 2b,d). The positive space charge remains in the sample after all annealing steps. However, its absolute value is changing.

Focused on the electric-field profiles and the space charge measured with negative polarity on the indium electrode (cathode) shown Figure 2a,b, respectively, the gradual annealing treatment resulted in a decrease in the positive space charge when compared to the as-grown state (from $8\times 10^{-10}$ C to $2\times 10^{-10}$ C, which corresponds to the charge density $1.1\times 10^{11}\;{\mathrm{cm}}^{-3}-2.7\times 10^{10}\;{\mathrm{cm}}^{-3}$ throughout the sample). There are considerable saw-tooth dependencies in Figure 2(a-insets),b that indicate a significant effect of the bias polarity during the annealing on the space charge. Here, the total space charge induced by annealing at negative and positive polarity forms the lower and upper envelope of its dependence shown in Figure 2b, respectively.

In the case of opposite polarity (In anode, Figure 2c), we observed the opposite effect of the applied bias at annealing on the values of the positive space charge (the blue circles follow a lower envelope line compared with the red squares in Figure 2d, which is an opposite behavior compared to Figure 2b). Furthermore, the positive total space charge at room temperature grew with the annealing steps. The electric field profile can be well approximated using a linear function in all cases reflecting a nearly constant distribution of the space charge in the volume of the sample.

In summary, the profiles of the internal electric field and the total space charge density evaluated at room temperature are influenced by both the temperature and polarity of the applied bias during annealing.

### 3.2. I–V Measurements

**Figure 3:**
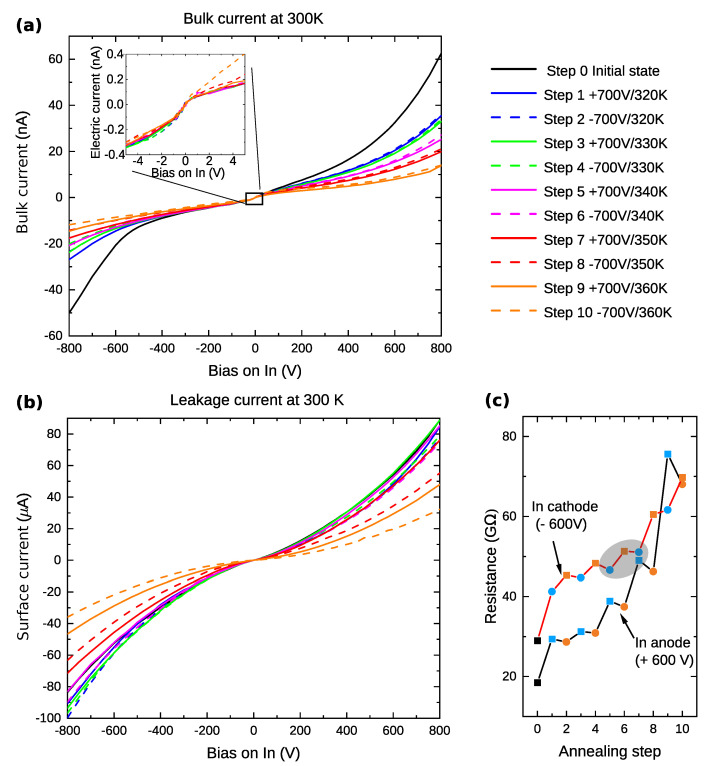
I–V characteristics of the bulk current showing the dependency on the annealing step (**a**). I–V characteristics of the leakage surface current (**b**). Bulk resistance evolution at ±600V (**c**). Here, the data points from grey area were later used for detailed analysis of the physical model in Section 3.3.

The I–V characteristics at room temperature after each annealing step are shown in Figure 3. The bulk current and leakage surface currents were measured separately.

**Figure 4:**
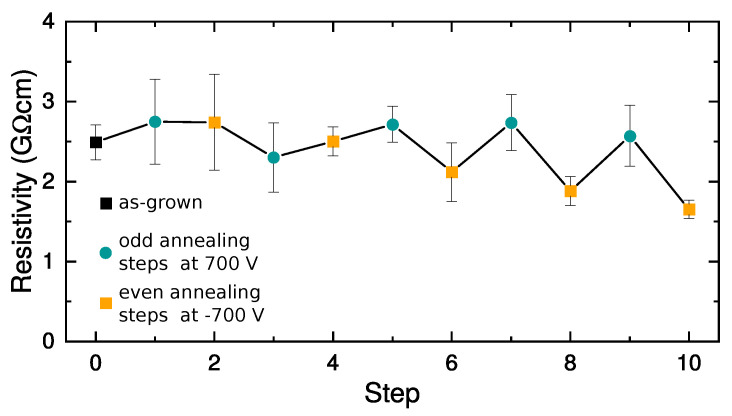
Sample specific resistivity at 300 K evaluated from the slopes of bulk I–V curves at low bias up to ±200mV.

Let us focus now on the bulk current, which is presented in Figure 3a. Firstly, we analyzed the material bulk specific resistivity ρ from the I–V characteristics around 0 V (inset of Figure 3a) using Ohm’s law I=VS/(ρl), where *S* is the contact area and *l* is the distance between contacts. The calculated sample’s specific resistivity depicted in Figure 4 shows a decrease with increasing annealing temperature. This decrease is smaller when the In contact is positively biased during annealing. After the final annealing step at 360 K (−700 V), the specific resistivity slightly decreased to $1.6\times 10^{9}$ Ωcm from its initial value of $2.5\times 10^{9}$ Ωcm before the first annealing step.

When the bias is increased above approx. ±1 V in both polarities (inset of Figure 3a), the slope of the I–V characteristics is smaller than at biases around 0 V. The characteristic has an S-shape character, indicating the depletion of free carriers in the bulk.

It is apparent that the resistance at high biases strongly depends on the applied annealing step. We specifically chose the resistance at ±600 V (Figure 3c) for further analysis and development of the model explaining the experimental data.

### 3.3. Physical Model

The most-important experimental results plotted in Figure 2 and Figure 3 are summarized in Table 1, which shows the evolution of the space charge and resistance R=V/I of the sample (evaluated at +600 V) induced by the annealing step. Double and single arrows correspond to the large and small change, respectively. We can identify the important correlation of the charge and resistance changes. When the resistance grows, the space charge decreases. When the resistance slightly decreases, the space charge increases. For the explanation of these phenomena, we must analyze the mechanisms responsible for the sample’s charging.

In semi-insulating semiconductors, both electrons and holes participate in the transport. The positive space charge formed in the biased semiconductor may be, in general, induced either by an injecting anode or a blocking cathode. Injecting an anode is increasing the concentration of holes that can be trapped at deep levels. This way a positive space charge in the bulk can be formed. Another possibility to form a positive space charge is to use a blocking cathode. In this case, electrons are depleted in the bulk, and an uncompensated fixed space charge is present in the volume of the sample. In our case, we may reject the injection of holes. The I–V characteristics displayed in Figure 3a clearly show the S-character proving the blocking contact. Moreover, the almost perfectly linear profiles of the electric field in Figure 2 sign the charging by carrier depletion. On the other hand, the carrier injection would produce curves deviating from linear profiles as predicted by the theory of the Space Charge Limited Currents [23,24]. Consequently, the blocking cathode may be considered as the primary attribute responsible for the positive space charge formation in the sample before annealing—step 0. Since the same features are observed for both positive and negative biasing, we may conclude that both contacts, Au and In, act in the same way.

**Figure 5:**
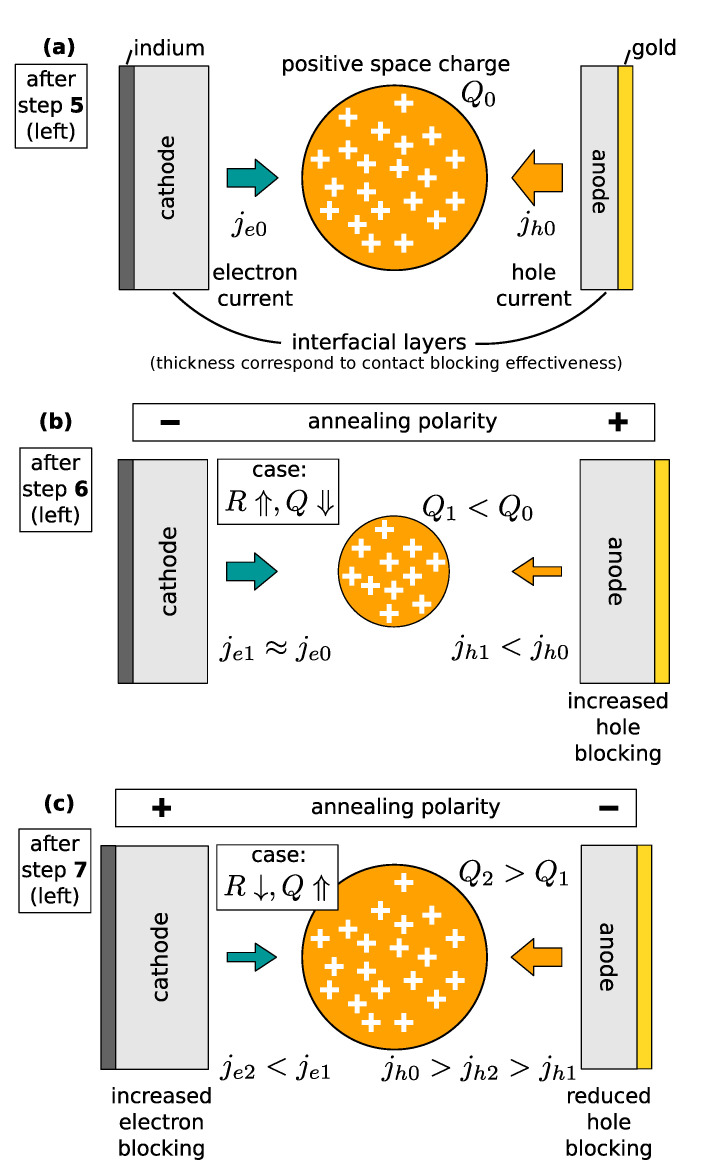
(**a**) A simple scheme of processes leading to the formation of positive space charge in the initial state before annealing. (**b**) A scheme after an annealing step, when the resistance increases and, at the same time, the positive space charge decreases. (**c**) A scheme after an annealing step when the resistance decreases and the space charge increases.

Let us now focus on the evolution of the resistance and space charge with the annealing steps—Figure 2c,d and Figure 3c and Table 1. We can see that the annealing steps resulting in the resistance increase are accompanied by a space charge reduction. This situation may be then unambiguously explained as the enhancement of the blocking character of the anode inducing the hole depletion. The electrical current is smaller and the resistance is higher due to the reduction of the hole component. At the same time, a smaller hole current results in a smaller hole trapping at deep levels. This way the positive space charge in the bulk is reduced. A scheme representing the above-mentioned processes is shown in Figure 5. Figure 5a,b represent the conditions in the sample after annealing steps 5 and 6, respectively. A scheme representing the processes leading to this situation is presented as a change between Figure 5a,b that represents the conditions in the sample after annealing steps 5 and 6, respectively.

The interpretation of the effect of the annealing steps resulting in the increase in the positive space charge is more complex. The increased cathode blocking or reduced anode blocking may result in the desired effect. That would, however, contradict the observed nearly unchanged specific resistivity evaluated from the slopes of the I–V characteristics at a bias close to 0—Figure 4. We thus conclude that this situation can be explained by the parallel activity of both mechanisms when the increased resistance caused by enhanced cathode blocking is compensated by reduced resistance due to lowered anode blocking. A scheme describing this situation is presented in Figure 5c, and it corresponds to the effect of annealing step no. 7.

### 3.4. Numeric Simulations

We tested the validity of the presented physical model by numerical simulation illustrating the oscillatory character of the charge and resistance shown in Figure 2b,d and Figure 3c, respectively. The calculations were done with the homemade code [23,25] solving the drift-diffusion and Poisson’s equations. We defined the defect structure of the bulk by trial parameters: Fermi energy $E_{F}=E_{c}-0.725$ eV, one electron trap with the density $N_{te}=3\times 10^{11}$ cm${}^{-3}$ localized below $E_{F}$ at the energy $E_{te}=E_{c}-0.79$ eV, and one hole trap above $E_{F}$ with the density $N_{th}=3\times 10^{11}$ cm${}^{-3}$ at the energy $E_{th}=E_{c}-0.66$ eV. Since we investigated only the steady state here, the respective capture cross sections cannot be determined [23]. The effect of the annealing was simulated by tuning transfer rates γ defining the source of carriers in both contacts [23]. We used flat bands at the simulations since the information on the band bending is not accessible. Besides, the respective band bending may be compensated by appropriate tuning of the transfer rates so that it does not disable the simulation.

Defining the source of the charge on contacts through the transfer rates γ represents the simplest option to introduce the model of contact barriers to the theory. It allows us to easily demarcate the maximum particular current conforming simultaneously to the zero electric current at zero bias. Evidently, a realistic model of contacts would have to be described by a much more complex theory. For such specification, we do not have enough experimental data, and the detailed contact properties investigation was not the primary aim of the research. The visualization of the fitted γ is more convenient in the inverted form $\gamma^{-1}$ since it corresponds to the blocking character of the interface barriers.

**Figure 6:**
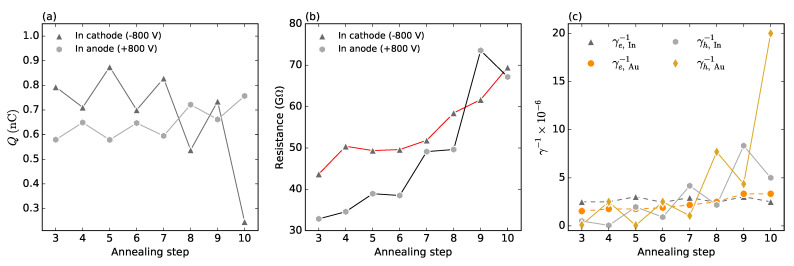
Numeric simulations. Total space charge (**a**) corresponding with experimental data shown in Figure 2b,d. Resistance at high voltage (**b**) corresponding with experimental data shown in Figure 3c. Inverted transfer rates for electrons ($\gamma_{e}^{-1}$) and holes ($\gamma_{h}^{-1}$) at both electrodes (In and Au) (**c**).

We plot in Figure 6a,b the results of the simulations of the charge and resistance, respectively. It is seen that the evolution of both quantities truly depicts the experimental data in Figure 2b,d. Corresponding inverted transfer rates $\gamma^{-1}$ are presented in Figure 6a. We can see that, in agreement with the suggested model, the polarization is more ruled by the evolution of barriers blocking holes (solid connecting lines). In case of electrons, the effect is less distinctive (dashed connecting lines). Nevertheless, the evolution of barriers blocking electrons has a dominant effect on the space charge tuning. In the negative electric field (see Figure 2b), the electron barrier at the In contact remains stable, and the ascending barrier of holes on Au leads to the reduction in the polarization. Oppositely, the electron barrier on Au contact grows similarly as the hole barrier at the In contact, which results in the stable polarization in spite of the enhanced resistance of the detector. Although the fit shows weak deviations from the experiment, the principal features were undoubtedly depicted. The better fit would need a comprehensive optimization involving both the defect structure and the surface barrier parameters.

### 3.5. Interpretation

The effects of low temperature annealing up to 360 K of the biased semi-insulating CZTS can be summarized as the following: The bulk material resistivity remains practically unchanged, while major changes affecting the electronic properties of the detector, such as the total space charge and dark current at high voltage bias (therefore, the resistance at ±600V) under which the detectors work by default, are associated with the development of surface states under the electrodes. During annealing, when the particular electrode is positive, its ability to block both types of carriers increases. If the electrode during annealing is negative, its ability to block holes decreases, while its ability to block electrons does not change. Observed changes show signs of universality because the effects do not depend on whether the electrode is indium or gold. By suitable tuning and a combination of conditions of low-temperature annealing under bias (temperature and polarity) and polarity used during the detector operation, its resistance can be significantly increased, and the effect of positive-space-charge accumulation on the traps can be reduced. In addition, it appears advantageous to anneal the sample at a temperature of at least 350 K, at which there is a significant reduction in the surface current, as can be seen in Figure 3b.

We assumed that the modification of the barriers at the contacts after low-temperature annealing could be explained by the development of an insulation layer between the semiconductor and metal electrodes. The difference between an expected I–V behavior based on the work function model and our observations can be attributed to a thin interfacial layer between the semiconductor and metallic electrodes. Although there is no source of oxygen during the annealing and characterisation processes, we expect the presence of a-few-nm thick oxide (TeO${}_{2}$) layer at the CZTS surface before contact deposition, which could slightly develop (change its thickness vs. concentration) during annealing and therefore affect the barrier’s electronic properties. We reported the initial presence of a 2.8 nm thick TeO${}_{2}$ layer at the CZTS surface with a concentration of 66% recently in the [16] estimated by ellipsometry measurements and analysis performed on the sample with the similar surface treatment. If there is a significantly thick interfacial layer with a reasonably high density of states, the Fermi level is pinned by the surface states, and the barrier height is independent of the metal workfunction, and it is given by the surface properties as mentioned in the [26].

## 4. Conclusions

We investigated the influence of low-temperature annealing under an applied bias on the basic electrical properties of the detector-grade material CdZnTeSe. We treated the CZTS sample with temperatures in the range where detectors normally work from 320 K to 360 K, and with a normal detector bias voltage (±700 V). We measured the internal electric field profiles and I–V characteristics after each annealing step. We observed that the profiles of the internal electric field and the total space charge density evaluated at room temperature are influenced by both the temperature and polarity of the applied bias during annealing. We analyzed the I–V characteristics measured at room temperature after each annealing step and evaluated the specific resistivity of the bulk and resistance at ±600 V. We could identify correlations between the charge and resistance changes. We explained the experimental data by a qualitative model based on the modification of the contact barriers by annealing. Low-temperature annealing of the contacts under bias can be used to modify the contact properties.

## Figures and Tables

**Table 1:** Overview of effects after the low-temperature annealing steps of biased CZTS sample. Change in the resistance *R* while biased at 600 V and the total space charge *Q*. Measured for both polarities at 300 K. Upwards (downwards) double arrow means a significant increase (decrease) in the value, while the downwards single arrow is a less-significant decrease.

	Odd Annealing Steps		Even Annealing Steps	
	Indium as Anode (+)		Indium as Cathode (−)	
Characterization:	*R* at 600 V:	*Q*:	*R* at 600 V:	*Q*:
Indium as anode (+)	⇑	⇓	↓	⇑
Indium as cathode (−)	↓	⇑	⇑	⇓

## Data Availability

Data collected through the research presented in the paper are available on request from the first and the corresponding authors.
